# Editorial: Interplay of plant volatiles in enhancing immunity and sustainable pest management

**DOI:** 10.3389/fpls.2026.1847766

**Published:** 2026-04-24

**Authors:** Islam S. Sobhy, Frédéric Francis

**Affiliations:** 1 School of Natural Sciences, University of Chester, Chester, United Kingdom; 2 School of Biosciences, Cardiff University, Cardiff, United Kingdom; 3 Functional and Evolutionary Entomology, University of Liège, Liège, Belgium

**Keywords:** herbivore, induced defense, integrated pest management, plant disease, plant immunity, plant volatiles, sustainable agriculture, tritrophic interactions

Volatile organic compounds (VOCs) have emerged as pivotal mediators of plant physiology and ecology, challenging their traditional characterization as mere metabolic by-products (Dudareva et al., 2013). These compounds function as chemical signals that coordinate plant defense responses and mediate ecological communication across multiple trophic levels, offering promising avenues for sustainable agricultural innovation (Turlings and Erb, 2018). The thirteen contributions compiled in this Research Topic advance both mechanistic understanding and practical applications of VOC-mediated plant protection strategies (**Figure 1**), building upon previous insights into the multifaceted roles of plant volatiles in agricultural ecosystems (Sobhy et al., 2022a).

**Figure 1:**
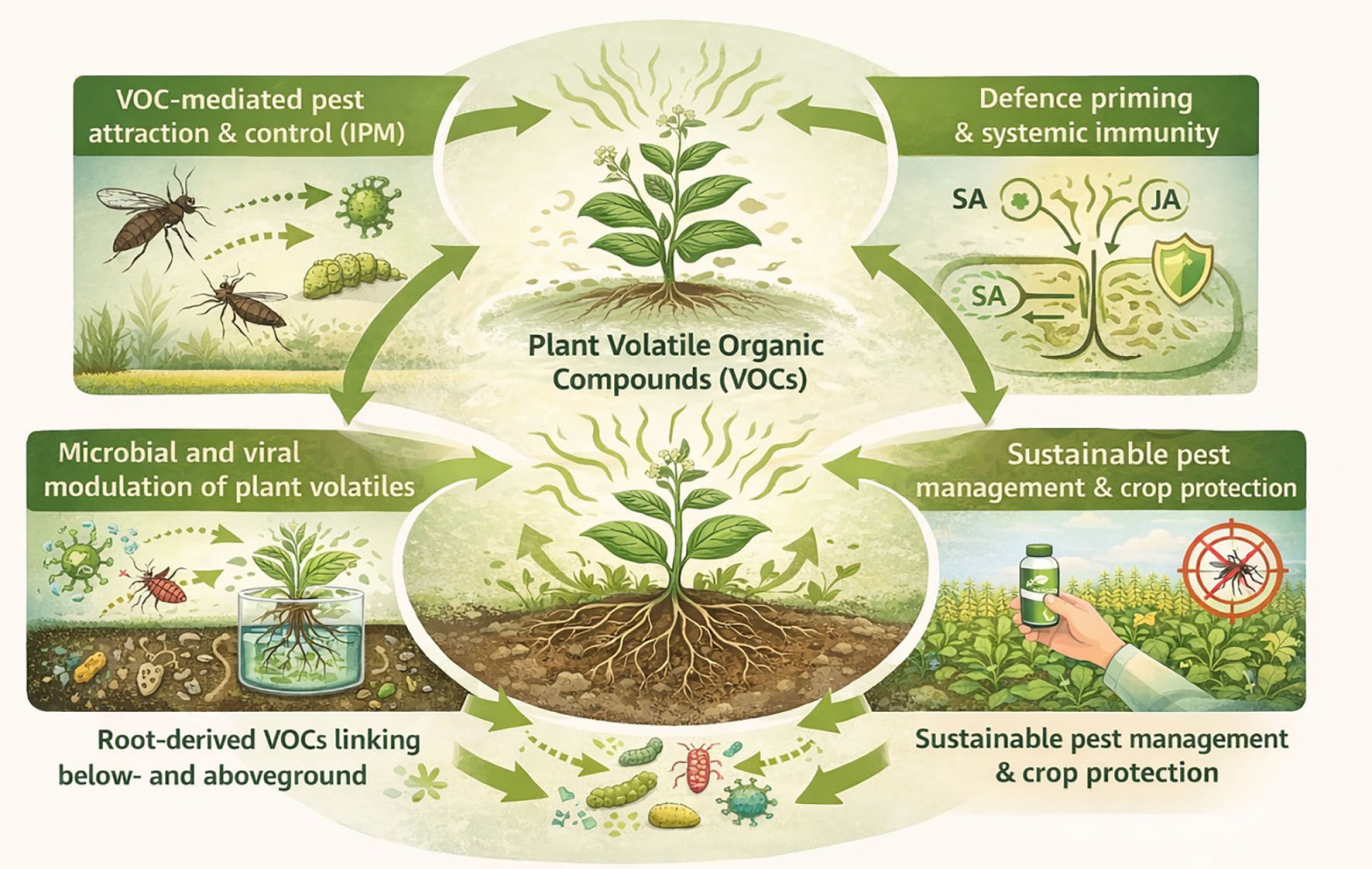
Schematic overview of the multifunctional roles of plant volatile organic compounds (VOCs), as discussed in this Research Topic, in plant defense and sustainable pest management. VOCs act as central signals that mediate tritrophic interactions, enhance parasitoid attraction, and influence herbivore behavior. They also prime plant immunity *via* key defense pathways (e.g. SA and JA) and facilitate belowground–aboveground communication through root-derived signals. Microbial and viral interactions further modulate VOC emissions, collectively supporting integrated and sustainable crop protection strategies.

## VOCs as attractants for integrated pest management

The manipulation of VOC emissions and how the insects perceive these altered VOC bouquets during host recognition and location represent currently a cornerstone of innovative integrated pest management (Bruce and Pickett, 2011; Sobhy et al., 2014; Zhou and Jander, 2022). In this context, Tabanca et al. demonstrated a compelling dual functionality of essential oil constituents from *Artemisia absinthium*, revealing that both monoterpenes α-thujone and β-thujone not only attract male Mediterranean fruit flies (*Ceratitis capitata*) but also exhibit potent toxicity against Caribbean fruit flies (*Anastrepha suspensa*). Such a bifunctional characteristic (i.e., combining attractant properties with insecticidal activities) highlights the potential for plant-derived volatiles to be integrated into sustainable pest management strategies that reduce reliance on synthetic pesticides. Similarly, methyl benzoate (MeBA), a common floral aromatic VOC, exhibits both insecticidal activity and attractant properties in several pest species, supporting such dual VOC Functions (Zhao et al., 2022).

In another article, Song et al. elucidated the olfactory mechanisms underlying the noctuid *Athetis dissimilis* responses to plant VOCs, identifying the green leaf volatiles (GLVs) trans-2-hexenal, *cis*-3-hexen-1-ol, and trans-2-hexen-1-ol as key compounds eliciting strong electroantennogram responses. Most promising was the finding that males exhibit higher sensitivity to these GLVs, associated with differences in odorant-binding protein expression. A similar pattern has been reported in *C. capitata*, where mated males exhibit higher sensitivity to host-plant fruit and leaf headspace volatiles than females (Sollai et al., 2020). These findings emphasize the importance of understanding sex-specific chemosensory mechanisms when developing VOC-based attractants and pest control strategies for effective field implementation.

The specificity of VOC-mediated tritrophic interactions is further highlighted by Sion et al., who investigated how egg parasitoids *Trissolcus japonicus* and *Trissolcu basalis* utilize volatile cues from stink bug egg masses for host location. The identification of γ-butyrolactone and β-funebrene in the headspace of *Nezara viridula* eggs, coupled with differential parasitoid responses to egg age, reveals the chemical complexity underlying host-parasitoid dynamics and offers practical applications for optimizing biological control programs.

## Defense priming and systemic immunity

VOCs function not only as external signals but also as internal priming agents that enhance plant immunity against herbivores (Conrath et al., 2015). In this regard, Ortells-Fabra et al. provided compelling evidence that synthetic herbivore-induced plant volatiles (HIPVs) activate defense pathways in citrus rootstocks. Notably, the GLVs (*Z*)-3-hexenyl propanoate and (*Z*)-3-hexen-1-ol consistently activated both salicylic acid and jasmonic acid pathways while simultaneously repressing *CsPUB21*, a gene associated with huanglongbing susceptibility. This dual mode of action (i.e., enhancing plant immune signaling while reducing the expression of a key susceptibility gene), a mechanism also demonstrated earlier in hybrid poplar by Frost et al. (2008), represents a sophisticated defense strategy with immediate practical applications for citrus disease management.

The genotypic variation in volatile responsiveness, as demonstrated across the four citrus rootstocks, emphasized that effective VOC-based priming strategies must account for genetic background. This principle is further reinforced by Markovic et al., who developed a trait-based laboratory method to identify cereal cultivar pairs capable of reciprocal volatile priming. Over three years of field trials, cultivar mixtures with two-way volatile interactions significantly reduced aphid infestation without compromising yield. These findings lend support to the practical potential of volatile-mediated plant–plant communication as a sustainable pest management strategy.

## Belowground chemical signaling and rhizosphere dynamics

The rhizosphere represents a frontier in VOC research, where root-derived signals mediate complex plant-microbe (Sharifi et al., 2022) and plant-plant interactions (Huang et al., 2019). Cascone et al. identified three lipophilic volatile molecules (i.e., 1-octen-3-ol, sulcatone, and sulcatol) in root exudates of aphid-infested *Vicia faba* plants that function as defense elicitors. When applied to intact plants via hydroponic systems, these compounds significantly enhanced plant attractiveness to the aphid parasitoid *Aphidius ervi*, establishing a direct link between belowground chemical communication and aboveground tritrophic interactions. Similar patterns were reported by Guerrieri et al. (2002), who found that *V. faba* plants exposed to hydroponic solution from aphid-infested plants became significantly more attractive to *A. ervi*. These findings highlight the importance of rhizosphere chemical signals in driving aboveground tritrophic interactions.

Methodological advances are also enhancing our ability to study such rhizosphere signals. To this end, Halane et al. introduced coated blade solid-phase microextraction for non-invasive, temporal metabolomic profiling of root exudates, enabling continuous monitoring of metabolite fluctuations without disrupting rhizosphere integrity. This would overcome a key limitation of traditional destructive sampling methods.

## Host selection and behavioral ecology

Understanding how herbivores perceive and respond to plant volatile profiles is essential for predicting pest pressure and developing deterrent strategies (Zhou and Jander, 2022). In this vein, Chen et al. reveal that the tea leafhopper *Empoasca onukii* exhibits differential behavioral preferences toward VOCs from three tea species, with attraction positively correlated with hexanol, linalool, and geraniol content. Transcriptomic analysis further identified nine key genes regulating biosynthesis of these volatiles, providing molecular targets for engineering pest-resistant cultivars.

Expanding this repertoire, Bian et al. screened fifteen native plant species from Xinjiang, China, identifying six with significant repellent activity against cotton aphids. GC-MS analysis and electroantennogram testing revealed fourteen effective repellent compounds, with volatiles from *Rhaponticum repens*, *Karelinia caspia*, *Launaea polydichotoma*, and *Brassica rapa* showing particular promise for eco-friendly aphid management in cotton systems.

## Microbial modulation of plant volatiles

Endophytic and rhizosphere microorganisms profoundly influence plant volatile emissions, adding another layer of complexity to VOC-mediated defense (Wilberts et al., 2022). In this article collection, Yang et al. demonstrate that the endophytic fungus *Pyrenochaeta nobilis* exhibits potent antagonistic activity against *Botrytis cinerea* in tomato, with fermentation filtrates achieving complete pathogen growth inhibition *in vitro* and significant disease reduction in planta. Genomic characterization, including carbohydrate-active enzymes and secondary metabolite biosynthetic gene clusters, provides mechanistic insights into fungal-mediated plant protection and informs future biocontrol optimization.

Plant viruses have evolved sophisticated strategies to manipulate host volatile emissions for their own transmission advantage (Blanc and Michalakis, 2016). In this regard, Li et al. demonstrate that Pepper veinal yellows virus, via its movement protein P4, manipulates host volatile biosynthesis to simultaneously attract both aphid vectors and non-vector whiteflies.

This co-attraction strategy (i.e., enhancing certain volatiles while suppressing others) facilitates viral replication through increased trans-zeatin accumulation in co-infested plants, revealing the ecological complexity of virus-vector-plant interactions with broad implications for disease epidemiology in agricultural systems where multiple insect species coexist.

## Flavonoids as multifunctional defense metabolites

Beyond airborne chemical signaling, other secondary metabolites contribute to plant defense through complementary mechanisms (Erb and Kliebenstein, 2020). For example, Zhang et al. demonstrate that seven flavonoids (e.g., naringenin, apigenin, and kaempferol) effectively suppress cotton aphid populations by deterring host settling, reducing phloem feeding, and decreasing reproductive rates, establishing them as promising eco-friendly alternatives to synthetic insecticides.

## Future directions and translational potential

The collective insights from these contributions elucidate multiple pathways for translating VOC research into agricultural practice. The mechanistic understanding of volatile biosynthesis, perception, and ecological function provides a foundation for engineering crop varieties with optimized volatile profiles that enhance natural pest suppression while maintaining or improving agronomic performance (Bruce et al., 2015). The demonstrated effectiveness of synthetic volatiles in priming plant defenses suggests opportunities for developing novel crop protection products that activate endogenous immunity rather than directly targeting pests (Mitchell et al., 2016).

Emerging technologies for non-invasive metabolomic profiling and high-throughput volatile phenotyping will accelerate the discovery of novel bioactive compounds and facilitate breeding programs targeting desirable volatile traits (Hall et al., 2022). Integration of chemical ecology with microbiome engineering presents exciting possibilities for developing microbial inoculants that modulate plant volatile emissions to enhance pest resistance and stress tolerance (Tronson and Enders, 2025). The successful field validation of volatile-primed cultivar mixtures and/or companion crops demonstrates that laboratory discoveries can be translated into practical cropping systems, providing a roadmap for future implementation studies (Dahlin et al., 2018; Sobhy et al., 2022b).

The advances compiled in this Research Topic demonstrate that volatile-based strategies are no longer speculative concepts, but evidence-based tools poised to transform sustainable agriculture. By harnessing VOCs for eco-friendly pest management alongside microbial manipulation and tritrophic interactions, we can develop resilient, high-performing cropping systems that reduce dependence on synthetic pesticides while maintaining food security in an era of environmental change.
